# Supplementary material for: A new computational model illuminates the extraordinary eyes of Phronima
Source: PLoS Comput Biol. 2022 Oct 17;18(10):e1010545. doi: 10.1371/journal.pcbi.1010545 (PMC9576097; doi:10.1371/journal.pcbi.1010545)
Supplement: S1 Fig — (PDF) [file pcbi.1010545.s001.pdf]

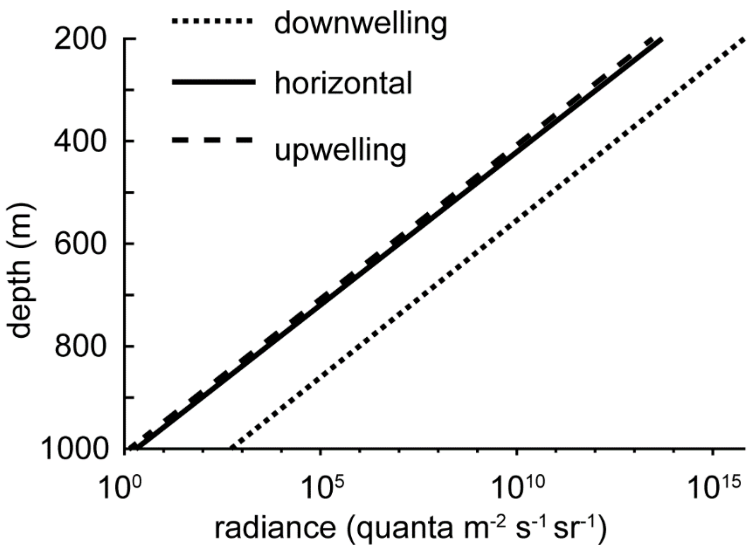

S1 Fig. The radiance of light in the equatorial Pacific at 1005 hrs seen from different viewing directions at different depths (data from [1]).

References

1. Johnsen S, Widder EA, Mobley CD. Propagation and perception of bioluminescence: factors affecting counterillumination as a cryptic strategy. The Biological Bulletin. 2004;207(1):1-16.
