# Supplementary material for: A new computational model illuminates the extraordinary eyes of Phronima
Source: PLoS Comput Biol. 2022 Oct 17;18(10):e1010545. doi: 10.1371/journal.pcbi.1010545 (PMC9576097; doi:10.1371/journal.pcbi.1010545)
Supplement: S2 Fig — Each column shows the effects on the maximum detection distance of increasing or decreasing, by a factor of two, the: (A) facet diameter, (B) quantum efficiency or integration time, and (C) acceptance angle of the ommatidium. The thick black solid line in each figure shows the result from the average medial eye parameters taken from our Phronima specimens (Table 2). The thinner black lines bounding the shaded areas show the result of decreasing (dark grey) or increasing (light grey) the parameters by a factor of two. (PDF) [file pcbi.1010545.s002.pdf]

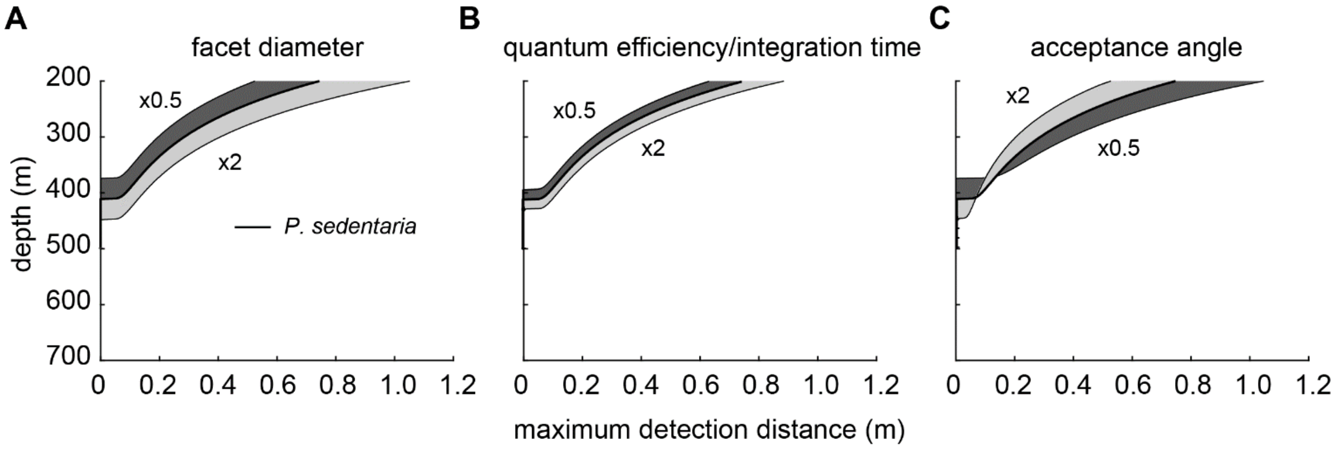

S2 Fig. The effects of visual parameters on modelled detection distances of the extended dark object with 0% transparency against downwelling radiance across depths. Each column shows the effects on the maximum detection distance of increasing or decreasing, by a factor of two, the: (A) facet diameter, (B) quantum efficiency or integration time, and (C) acceptance angle of the ommatidium. The thick black solid line in each figure shows the result from the average medial eye parameters taken from our *Phronima* specimens (Table 2). The thinner black lines bounding the shaded areas show the result of decreasing (dark grey) or increasing (light grey) the parameters by a factor of two.
