# Supplementary material for: A new computational model illuminates the extraordinary eyes of Phronima
Source: PLoS Comput Biol. 2022 Oct 17;18(10):e1010545. doi: 10.1371/journal.pcbi.1010545 (PMC9576097; doi:10.1371/journal.pcbi.1010545)
Supplement: S3 Fig — (A) Search volume for point source initially decreases but after Δρ:Δφ of three the search volume increases as the overlap increases. (B) The search volume for extended luminous object has the same trend as point source except at depth of 200m where detection distance always increases as the overlap increases. (C) The search volume for extended dark object with 50% transparency always increases with overlap at depths of 300 m and 400 m. At 200 m depth, initially there is a slight decrease in search volume. However, after Δρ:Δφ of three the search volume increases as the overlap increases. The number of ommatidia summating in a single channel was calculated as a function of Δρ:Δϕ ratio[10]. Search volumes were calculated using the derivations shown in the S3 Appendix. The acceptance angle was taken from the medial eyes of Phronima, 3.9° (Table 2), and we varied the interommatidial angle (from 0.26 to 3.9°) to model different Δρ:Δϕ ratios. Dotted vertical lines show the calculations for the overlap measured in our Phronima specimens (Δρ:Δϕ = 2.4) and dashed vertical lines show the calculations for the overlap measured by Land [6](Δρ:Δϕ = 9.2). (PDF) [file pcbi.1010545.s003.pdf]

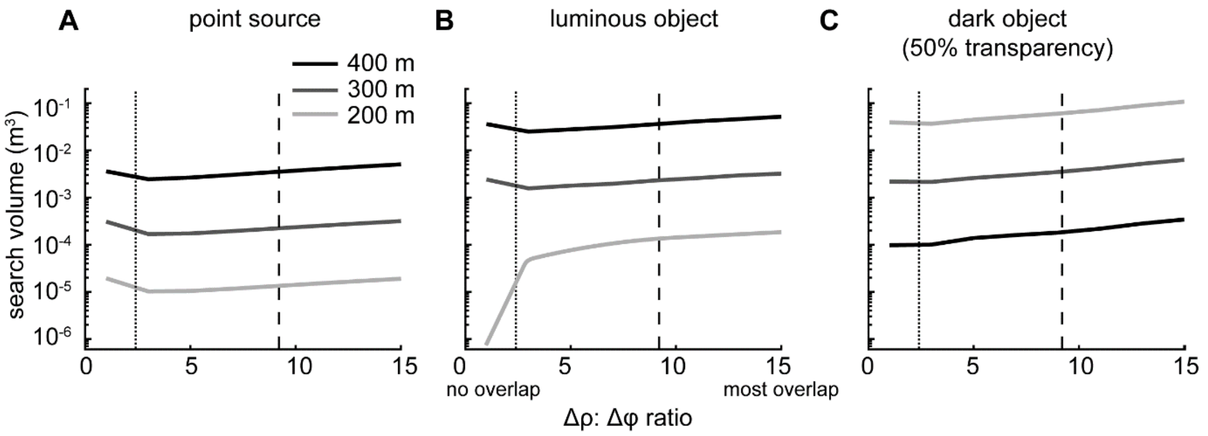

S3 Fig. The effect of receptive overlap between neighbouring ommatidia on search volume. (A) Search volume for point source initially decreases but after *Δρ:Δφ* of three the search volume increases as the overlap increases. (B) The search volume for extended luminous object has the same trend as point source except at depth of 200m where detection distance always increases as the overlap increases. (C) The search volume for extended dark object with 50% transparency always increases with overlap at depths of 300 m and 400 m. At 200 m depth, initially there is a slight decrease in search volume. However, after *Δρ:Δφ* of three the search volume increases as the overlap increases. The number of ommatidia summating in a single channel was calculated as a function of *Δρ:Δϕ* ratio[1] . Search volumes were calculated using the derivations shown in the S3 Appendix. The acceptance angle was taken from the medial eyes of *Phronima*, 3.9º (Table 2), and we varied the interommatidial angle (from 0.26 to 3.9º) to model different *Δρ:Δϕ* ratios. Dotted vertical lines show the calculations for the overlap measured in our *Phronima* specimens (*Δρ:Δϕ* =2.4) and dashed vertical lines show the calculations for the overlap measured by Land [2](*Δρ:Δϕ* 9.2).

References

1. Land MF. Optics of the eyes of Phronima and other deep-sea amphipods. Journal of Comparative Physiology A: Neuroethology, Sensory, Neural, and Behavioral Physiology. 1981;145(2):209-26.
2. Land MF. The eyes of hyperiid amphipods: relations of optical structure to depth. Journal of Comparative Physiology A: Neuroethology, Sensory, Neural, and Behavioral Physiology. 1989;164(6):751-62.
