# Supplementary material for: A new computational model illuminates the extraordinary eyes of Phronima
Source: PLoS Comput Biol. 2022 Oct 17;18(10):e1010545. doi: 10.1371/journal.pcbi.1010545 (PMC9576097; doi:10.1371/journal.pcbi.1010545)
Supplement: S1 Appendix — (PDF) [file pcbi.1010545.s004.pdf]

S1 Appendix. Derivation of Equation 1.8

Equation 2.4 from [1] shows that N_{bio} is:

$$N_{bio} = \frac{EA^2}{16r^2} e^{-\alpha r} q\Delta t. \quad \text{Eq. S1.1}$$

In order to find *r* we have rearranged Eq. S1.1 as follows:

$$r^2 e^{\alpha r} = \frac{EA^2 q\Delta t}{16N_{bio}} \quad \text{Eq. S1.2}$$

$$r e^{\frac{\alpha}{2}r} = \sqrt{\frac{EA^2 q\Delta t}{16N_{bio}}} \quad \text{Eq. S1.3}$$

and by multiplying both sides by $\frac{\alpha}{2}$ we have:

$$\frac{\alpha}{2} r e^{\frac{\alpha}{2}r} = \frac{\alpha}{2}\sqrt{\frac{EA^2 q\Delta t}{16N_{bio}}}. \quad \text{Eq. S1.4}$$

Assuming a dummy variable $x = \frac{\alpha}{2}r$, Eq. S1.4 becomes:

$$x e^{x} = \frac{\alpha}{2}\sqrt{\frac{EA^2 q\Delta t}{16N_{bio}}} \quad \text{Eq. S1.5}$$

and using the Lambert W-function (the inverse function of $f(x) = xe^x$) Eq. S1.5 will become:

$$x = W\left(\frac{\alpha}{2}\sqrt{\frac{EA^2 q\Delta t}{16N_{bio}}}\right). \quad \text{Eq. S1.6}$$

Substituting $\frac{\alpha}{2}r$ for *x* and simplifying the right side of Eq. S1.6 then gives:

$$\frac{\alpha}{2} r = W\left(\frac{\alpha A}{8}\sqrt{\frac{Eq\Delta t}{N_{bio}}}\right) \quad \text{Eq. S1.7}$$

which can be rearranged to find *r* as shown below.

$$r = \frac{2W\left(\frac{\alpha A}{8}\sqrt{\frac{Eq\Delta t}{N_{bio}}}\right)}{\alpha} \quad \text{Eq. S1.8}$$

References

1. Nilsson D-E, Warrant E, Johnsen S. Computational visual ecology in the pelagic realm. Philosophical Transactions of the Royal Society B: Biological Sciences. 2014;369(1636):20130038.
