# Supplementary material for: A new computational model illuminates the extraordinary eyes of Phronima
Source: PLoS Comput Biol. 2022 Oct 17;18(10):e1010545. doi: 10.1371/journal.pcbi.1010545 (PMC9576097; doi:10.1371/journal.pcbi.1010545)
Supplement: S2 Appendix — (PDF) [file pcbi.1010545.s005.pdf]

S2 Appendix. Derivation of the solid angels of extended dark object and background

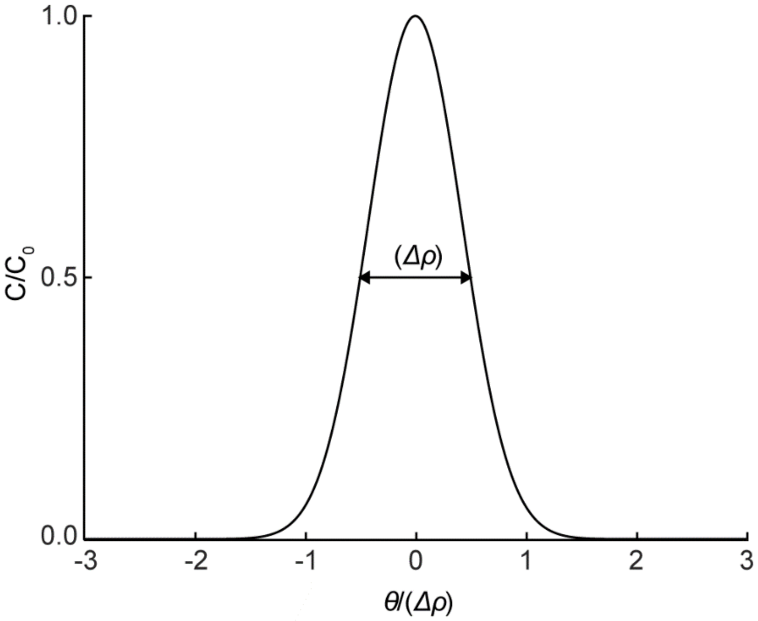

Fig A. The angular sensitivity profile of an ommatidium with a Full Width at Half Maximum equal to $\Delta\rho$.

If we assume the angular sensitivity profile of an ommatidium, about its long axis, follows a Gaussian function (Fig A in S2 Appendix), this can be described by the equation:

$$\frac{C}{C_0} = e^{(-M\theta^2)} \quad \text{Eq. S2.1}$$

where θ is the angle, in radians, between the axis of the ommatidium and a line to the centre of the distal tip of the ommatidial crystalline cone to a point in visual space.

M is then a scaling factor such that when:

$$\theta = \pm\frac{\Delta\rho}{2}$$

In other words, the Half Width at Half Maximum of the Gaussian is half of the ommatidial acceptance angle, $\Delta\rho$ (see Tables 1 and 2), or as the acceptance angle can be approximated by:

$$\frac{\Delta\rho}{2} = \frac{({}^{d}/_{f})}{2}$$

therefore:

$$\frac{1}{2} = e^{(-M(\frac{({}^{d}/_{f})}{2})^2)} \quad \text{Eq. S2.2}$$

thus:

$$M = \frac{4\ln 2}{({}^{d}/_{f})^2} \qquad \text{Eq. S2.3}$$

$$\frac{C}{C_0} = e^{(-4\ln 2\times(\frac{\theta}{({}^{d}/_{f})})^2)}. \qquad \text{Eq. S2.4}$$

The solid angle, in steradians, of the Gaussian profile from $\theta_1 to\ \theta_2$is then given by:

$$\Omega_i = \int_{\theta=\theta_1}^{\theta_2} \int_{\phi=0}^{2\pi} e^{(-4\ln 2\times(\frac{\theta}{({}^{d}/_{f})})^2)}\, \theta\, d\phi\, d\theta \qquad \text{Eq. S2.5}$$

Integrating over ϕ, the angle in the plane perpendicular to the ommatidial axis which ranges.$0 \leq \phi \leq 2\pi$:

$$\Omega_i = 2\pi \int_{\theta=\theta_1}^{\theta_2} e^{(-4\ln 2\times(\frac{\theta}{({}^{d}/_{f})})^2)}\, \theta\, d\theta. \qquad \text{Eq. S2.6}$$

Assuming a dummy variable $y = 4\ln 2 \times (\frac{\theta}{({}^{d}/_{f})})^2$

$$\Omega_i = 2\pi(\frac{({}^{d}/_{f})^2}{8\ln 2}) \int_{y=y_1}^{y_2} e^{-y}\, dy \qquad \text{Eq. S2.7}$$

$$\Omega_i = 2\pi\left(\frac{({}^{d}/_{f})^2}{8\ln 2}\right)(e^{-y_1} - e^{-y_2}). \qquad \text{Eq. S2.8}$$

If we want the solid angle of a full Gaussian we need to consider the sensitivity profile as a function of θ between θ_1 and θ_2 these angles being 0 and ∞ respectively. This yields $y_1 = 0$ and $y_2 = \infty$ therefore the solid angle of a full Gaussian profile will be:

$$\Omega_F = 1.133\ ({}^{d}/_{f})^2. \qquad \text{Eq. S2.9}$$

For full derivations of the solid angle of a Gaussian function see [1].

Because we assume a portion of Ω_F is covered by an extended dark object that is centred on the receptive field and has an angular size of 2θ, $y_1 = 0$ and $y_2 = 4\ln 2(\frac{\theta}{({}^{d}/_{f})})^2$, which gives us:

$$\Omega_o = 1.133\left(\frac{d}{f}\right)^2 (1 - e^{-4\ln 2 \times \left(\frac{\theta}{(d/f)}\right)^2}) \qquad \text{Eq. S2.10}$$

where Ω_o is the solid angle of the receptive field that is covered by the object. The remaining portion of the receptive field that is not covered by the object has limits of $y_1 = 4\ln 2 \times (\frac{\theta}{(d/f)})^2$ and $y_2 = \infty$ which yields a solid angle of:

$$\Omega_b = 1.133\left(\frac{d}{f}\right)^2 e^{-4\ln 2 \times \left(\frac{\theta}{(d/f)}\right)^2}. \qquad \text{Eq. S2.11}$$

References

1. Condon JJ, Ransom SM. Essential radio astronomy: Princeton University Press; 2016.
