# Supplementary material for: A new computational model illuminates the extraordinary eyes of Phronima
Source: PLoS Comput Biol. 2022 Oct 17;18(10):e1010545. doi: 10.1371/journal.pcbi.1010545 (PMC9576097; doi:10.1371/journal.pcbi.1010545)
Supplement: S3 Appendix — (PDF) [file pcbi.1010545.s006.pdf]

S3 Appendix. Derivation of the search volume, total acceptance angle of a single channel, and the size of the full visual field

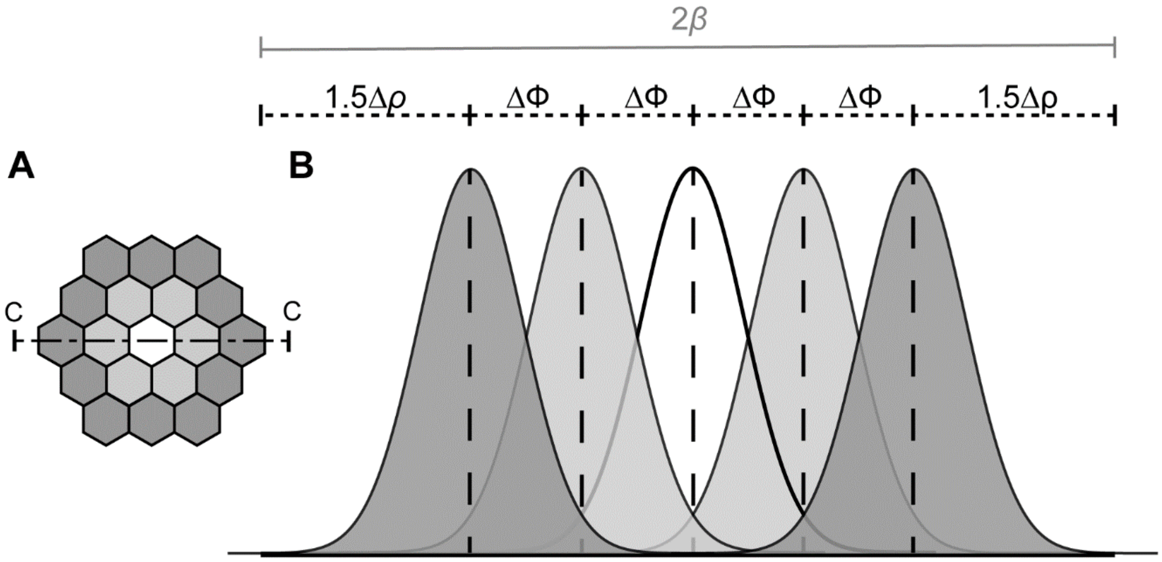

Fig A. Total acceptance angle of a single channel. (A) A hexagonal array representing the arrangement of ommatidia in a single channel comprised of three neighbourhood. Each neighbourhood represented by a different colour. (B) Total acceptance angle of a single channel (2β) is calculated assuming the Gaussian receptive field has a full width of approximately $3\Delta\rho$. The sensitivity functions of ommatidia are shown along the cross sections c-to-c in (A).

The search volume was approximated by calculating the volume of a spherical cone as follows:

$$V(\beta) = \frac{2}{3}\pi r^3(1 - cos\beta) \qquad \text{Eq. S3.1}$$

where r is the maximum detection distance and β is half of the summated acceptance angle (the total acceptance angle of the channel). To calculate β we assume the Gaussian receptive field has a full width of approximately $3\Delta\rho$. Therefore, the full acceptance angle of a single channel (Fig A in S3 Appendix) would be:

$$2\beta = 3\Delta\rho + 2n\Delta\varphi. \qquad \text{Eq. S3.4}$$

where n is the number of neighbourhoods that forms the channel excluding the central ommatidia (e.g. n=2 in S3 Appendix Fig A).

The size of visual field was also calculated by Eq. S3.4. For this purpose we calculated n for the total number of ommatidia in one eye and assuming that all 420 ommatidia in the eye forms a perfect hexagonal array similar to the one depicted in panel A of Fig A in S3 Appendix.
