# Supplementary material for: A new computational model illuminates the extraordinary eyes of Phronima
Source: PLoS Comput Biol. 2022 Oct 17;18(10):e1010545. doi: 10.1371/journal.pcbi.1010545 (PMC9576097; doi:10.1371/journal.pcbi.1010545)
Supplement: Attachment — Submitted filename: PCB_modelling_rejoinder_final.pdf [file pcbi.1010545.s008.pdf]

THE UNIVERSITY OF WESTERN AUSTRALIA

Dr. Zahra Bagheri

School of Biological Sciences, M092

Postdoctoral Researcher

The University of Western Australia,
35 Stirling Hwy, Crawley WA 6009

Tel +61 8 6488 3317

Email: zahra.bagheri@uwa.edu.au

CRICOS Provider Code: 00126G

Tuesday, 30 August 2022

Dear Professor Warrant:

On behalf of the authors, I would like to thank you for considering our article "A new computational model illuminates the extraordinary eyes of *Phronima*." for publication in Plos Computational Biology. We appreciate the reviewers' valuable comments and positive feedback, which we believe increased the impact of the manuscript. We have amended our manuscript in response to reviewers' comments. All these changes are highlighted in the manuscript. Please find below our detailed response to reviewers' comments.

Yours sincerely,

Zahra Bagheri

Reviewer 1

1- Line 54: I think you want "if they bioluminesce" or "if they emit bioluminescence" or "if they are bioluminescent" here.

This if fixed.

2-Line 79: Effects of the transparency of what? The answer becomes clear later, but I think you'll want to specify "effects of the transparency of extended objects" here.

This is added to the manuscript.

3-Line 90: I think "morphologically distinct" would be a better choice here than "spatially distinct".

We changed this in the manuscript.

4- Line 132: Check descriptions for panels A and B -- I think both are missing a few words.

This is fixed.

5- Methods: Please add a few more notes about where the methods developed by Nilsson et al end and the new methods begin. I appreciate the credit the current authors are giving to past authors, but I'm finding the dividing line between past and present to be a bit opaque.

We have clarified this in the manuscript (lines 172-175).

"We have adapted the equations from Nilsson et al. [9] for bioluminescent point sources and extended bright objects to account for the properties of compound eyes. We also developed a new model for detection of semi-transparent objects. In addition, we examined the effect of spatial summation on the discrimination thresholds of all tasks."

6- Figure 2 (and perhaps other figures): Please make sure all panels are cited in the text and that they are cited in the correct order. Unless this isn't a requirement for PLOS? I know it's a requirement for some journals.

Fixed as suggested.

7- Table 1: I very much appreciate the addition of Table 1 to the manuscript. It is quite helpful. Please double-check that all relevant terms and values are included here. I think Si (from Section 1.2) might be missing? Perhaps others are too?

We added the missing parameters to the table.

8- Lines 500-501: I'm not sure how to interpret the units here: "Extended objects were 1 cm m in diameter".

This typo is fixed.

9- Lines 587-588: If you want to test the hypothesis that animals with broader depth ranges have ommatidia with larger acceptance angles, perhaps compare the eyes of pelagic species to those of benthic species? Diel vertical migration makes me sceptical of published depth ranges for pelagic species, but I think one can make comparisons between pelagic and benthic species with a high degree of confidence.

We appreciate the reviewer's suggestion and are similarly skeptical of pelagic depth ranges because of migration, non-specificity of trawl data, and species complexes and we agree that would be the way to test our hypothesis. However, we think such a comparison is beyond the scope of the current paper and perhaps could be the focus of our future research.

10- Discussion (overall): It is curious to me that the authors do not cite or discuss the 2015 paper on the eyes of *Paraphronima gracilis* by Fergus, Johnsen, and Osborn. It also seems odd that the authors do not reference the 2021 paper by Lin et al on optic lobe organization in hyperiid amphipods. I'm not finding any statements in the Discussion that I want to debate, but I would like to see the Discussion updated to include recent papers on the eyes and brains of hyperiid amphipods, such as those by Fergus et al and Lin et al (there may be more, but these two came to mind immediately). Please note that I am not an author on either of these papers!

We have added Fergus et al. 2015 to our discussion (line 679-681).

"Fergus et al. [46] suggested that a hyperiid amphipod Paraphronima gracilis might use its unique discontinuous retinal configuration paired with neural adaptations for spatial summation via rows of ommatidia instead of within neighbourhoods."

However, since our paper focuses on the computational modelling of hyperiid amphipods vision rather than their brain we did not find Lin et al as a proper reference in our discussion.

Reviewer 2

1- The methods section outlining the many different use-cases of the Nilsson model they develop is too long and detailed with too many variables introduced, it makes it hard to tell how many different models there are and the differences between them. This needs a good clarifying overhaul. I suggest outlining an initial use of the basic model in detail, with a new schematic figure that illustrates the spatial relationships between the quantities and variables employed in the model. From there, each new use case can state briefly how the model was tweaked, what any additional underlying assumptions are, and simply state the calculation for sensitivity used downstream. The additional algebra and derivations could then be included in a longer supplementary methods section.

We appreciate the Reviewer's suggestion. Although we have used the base of Nilsson et al.'s model to account for properties of apposition compound eyes, our models are significantly different from theirs. Nilsson et al. do not provide any model for semi-transparent objects or spatial summation. Therefore, we do not believe focusing on explaining the basic model in detail would favour explaining our method since the readers can refer to the well explained model of Nilsson et al. As pointed out by both Reviewer 1 and the editor we made the effort to describe our methods clearly and thoroughly so it would be repeatable for readers and moving the included algebra and derivations to the supplementary could reduce the readability of the method section. Therefore, we did not make any significant changes to our explanation of models. However, we made some changes to our Figure 2, so our description of the method would be clearer and made a few edits to clarify methods as requested by Reviewer 1 (please see above).

2- Given the difficulty of working in the midwater, there are necessarily uncertainties about important quantities like integration time and quantum efficiency of these ommatidia. The authors find plausible reference values. Similarly, there is a pretty big disagreement between the degree of angular overlap between ommatidia they measured, and older data from Land. Given this uncertainty, and the interesting work they did to develop the Nilsson model for apposition compound eyes, I think this would be a far more interesting piece of work if they shifted the emphasis of the paper to explore the optimality of compound eyes for the different visual tasks they identify (point sources, extended bright objects, and extended dark objects). For example: what is the optimum angular overlap between ommatidia for viewing point sources at the peak of the organism's light/depth distribution?

And how different is that from either their or Land's observations? And are those observations more similar than different given the size and shape of the optimality landscape? Given the shape of this landscape, is *Phronima* more likely to be a visual specialist or generalist? I think this approach is more likely to provide insight about the specific evolved functions of *Phronima*'s eyes than the current approach of a parameter sweep in the face of significant parameter uncertainty.

In order to address the reviewer's comments, we have extended our analysis in an attempt to find an optimum overlap for detection of different targets. We calculated detection distances for different overlap; however, we did not find an optimum overlap. Because of the trade-off between detection distance and the size of visual field due to ommatidial overlap, we further looked at the search volume of the eye. However, we did not find any optimum overlap for search volume either. We amended our manuscript and included a new section in our results (lines 505-525):

"***The effect of ommatidial overlap on detection distances***

Increasing the overlap of neighbouring ommatidia by decreasing the interommatidial angle, increases detection distances (Figure 7A-C) but inevitably decreases the overall receptive field size of the target channel and the animal's overall field of view. This leads to a clear trade-off between detection distance and field of view (Figure 7D-F). This trade-off could be expected to lead to an optimum in the volume of water the animal can survey at any point in time since search volume is a function of both detection distance and visual field size. However, there is no optimum in the eye's search volume as a function of ommatidial overlap (Figure S3).

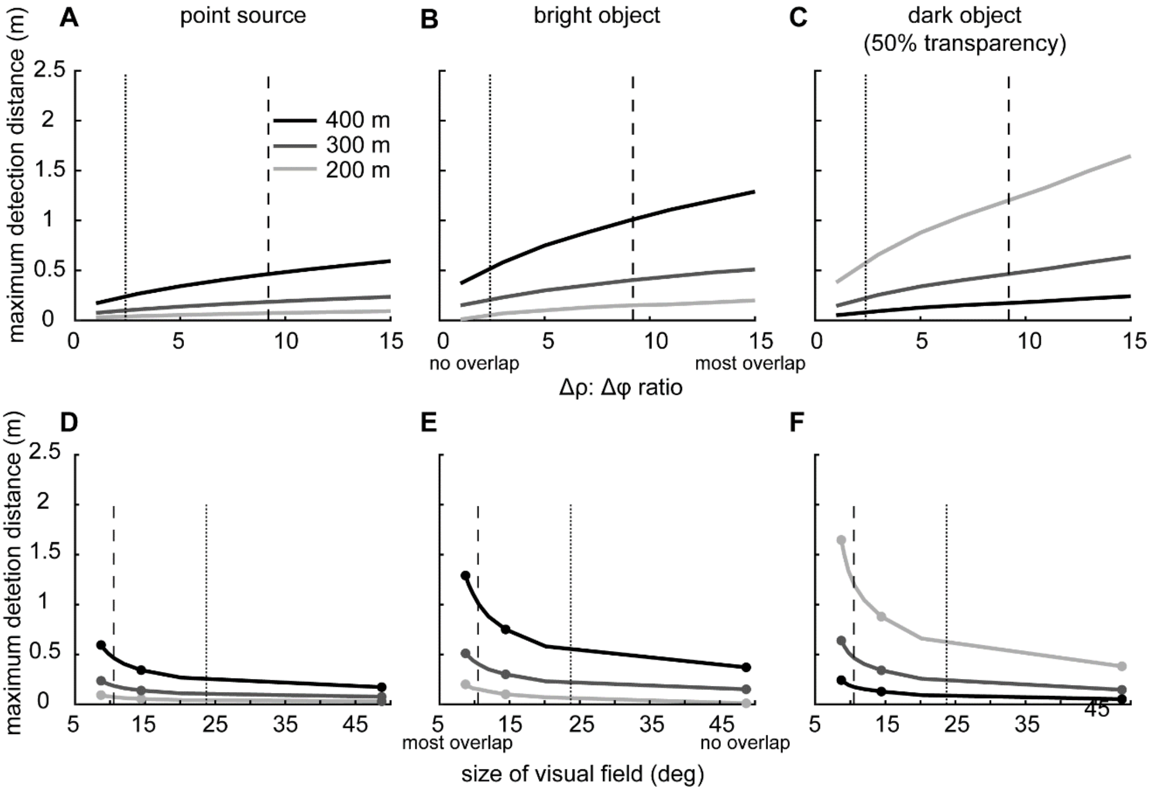

Figure 7. Effect of overlap of ommatidial receptive fields on detection distances and the size of visual field. (A-C) Increasing overlap always improved maximum detection distance for all targets. (D-E) However, there is a clear trade-off between detection distance and the size of field of view due to ommatidial overlap. Columns show the model results for the detection of the point source (A, D), the extended bright object (B, E), and the extended dark object (C, F). The number of ommatidia summating in a single channel was calculated as a function of Δρ:Δϕ ratio [10]. For overlaps greater than 16, there are not enough ommatidia within an eye of Phronima to from two distinct channel. The size of the field of view and the receptive field size of the target channel were calculated using the derivations shown in the Appendix S3. The acceptance angle was taken from the medial eyes of Phronima, 3.9º (Table 2), and we varied the interommatidial angle (from 0.16 to 3.9º) to model different Δρ:Δϕ ratios. Dotted vertical lines show the overlap measured in our Phronima specimens (Δρ:Δϕ =2.4) and dashed vertical lines show the overlap measured by Land [6] (Δρ:Δϕ 9.2). Small circles in (D-F) shows Δρ:Δϕ ratios of 1, 5 and 15. "

We included the results for search volume in the supplementary material:

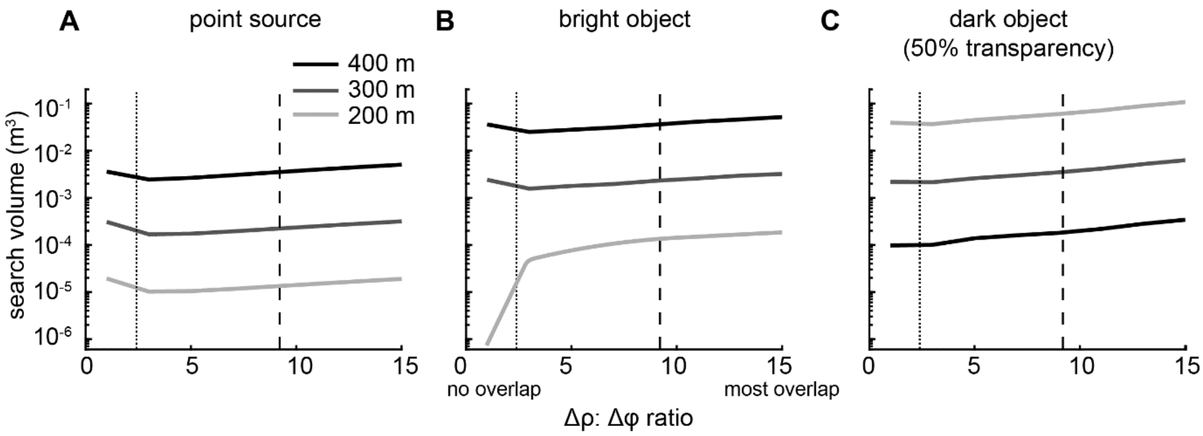

Figure S3. *The effect of receptive overlap between neighbouring ommatidia on search volume. (A) Search volume for point source initially decreases but after Δρ:Δφ of three the search volume increases as the overlap increases. (B) The search volume for extended bright object has the same trend as point source except at depth of 200mwhere detection distance always increases as the overlap increases. (C) The search volume for extended dark object with 50% transparency always increases with overlap at depths of 300 m and 400 m. At 200 m depth, initially there is a slight decrease in search volume. However, after Δρ:Δφ of three the search volume increases as the overlap increases. The number of ommatidia summating in a single channel was calculated as a function of Δρ:Δϕ ratio (Land, 1981). Search volumes were calculated using the derivations shown in the Appendix S3. The acceptance angle was taken from the medial eyes of Phronima, 3.9º (Table 2), and we varied the interommatidial angle (from 0.16 to 3.9º) to model different Δρ:Δϕ ratios. Dotted vertical lines show the calculations for the overlap measured in our Phronima specimens (Δρ:Δϕ =2.4) and dashed vertical lines show the calculations for the overlap measured by Land (1989) (Δρ:Δϕ 9.2).*

We also added a few sentences to the discussion discussing these results (lines 638-643).

"The overlap of the receptive fields of neighbouring ommatidia increases detection distances but comes at the cost of narrowing the animal's visual field (Figure 7). We did not find any optimum overlap of the receptive field for either detection distances or search volume (Figure 7 and Figure S3). The optimum solution most likely depends on species specific factors such as the cost functions for search distance and size of visual field, or what detection distances are relevant for animals of a certain size and speed. However, these factors are as yet unknown and our modelling did not provide any firm predictions."

However, we did not change our focus from understanding what the animals have and can see to what they would have with an optimum designed eye. Our main aim is to understand the function of the eyes, the trade-offs involved in arriving at the eyes as they are, and understanding the link between behaviours and eye form.

3- Another question that I am curious about. The Nilsson model relies heavily on the assumption of a Gaussian spatial sensitivity to incoming light for a single lens. This assumption was made in the context of camera-type eyes. The optics of the crystallin cones at the surface of *Phronima* ommatidia are very different. Is this assumption of Gaussian sensitivity profile still usable? The authors don't discuss this point. Is the Gaussian assumption appropriate here, or contrarily, is there any possible sensitivity distribution that would significantly change the model's results?

We agree that this is an interesting question. Unfortunately, we have no way of measuring the sensitivity profile of *Phronima*'s optics at this point. However, we do not believe this would change our main results and conclusions. If the sensitivity profile of *Phronima's* optics is closer to a square rather than Gaussian the detection distances would increase in all cases, however, it will not affect our conclusion that spatial summation would improve detection distances since the sensitivity of a pooling number of ommatidia would also increase with a more square profile. Given the effect of summation will not change, therefore, *Phronima*'s medial eye with spatial summation would still outperform the lateral eye. If the sensitivity profile of *Phronima's* optics has weaker tails than a Gaussian, we expect the detection distances would decrease in all cases but the argument regarding the effect of spatial summation and the performance of medial eye versus lateral eye still stands as before. Therefore, since we do not know the exact sensitivity profile of *Phronima*'s ommatidia, we believe assuming a Gaussian profile is an appropriate first step.
